# A touchdown nucleic acid amplification protocol as an alternative to culture backup for immunofluorescence in the routine diagnosis of acute viral respiratory tract infections

**DOI:** 10.1186/1471-2180-4-41

**Published:** 2004-10-25

**Authors:** Peter V Coyle, Grace M Ong, Hugh J O'Neill, Conall McCaughey, Dennis De Ornellas, Frederick Mitchell, Suzanne J Mitchell, Susan A Feeney, Dorothy E Wyatt, Marian Forde, Joanne Stockton

**Affiliations:** 1 Regional Virus Laboratory, Royal Hospitals Trust, Belfast, BT12 6BA, UK; 2 Bacteriology, Royal Hospitals Trust, Belfast, BT12 6BA, UK; 3 Enteric, Respiratory and Neurological Virus Laboratory, Health Promotion Agency, 61 Colindale Avenue, London NW9 5HT, UK

## Abstract

**Background:**

Immunofluorescence and virus culture are the main methods used to diagnose acute respiratory virus infections. Diagnosing these infections using nucleic acid amplification presents technical challenges, one of which is facilitating the different optimal annealing temperatures needed for each virus. To overcome this problem we developed a diagnostic molecular strip which combined a generic nested touchdown protocol with in-house primer master-mixes that could recognise 12 common respiratory viruses.

**Results:**

Over an 18 month period a total of 222 specimens were tested by both immunofluorescence and the molecular strip. The specimens came from 103 males (median age 3.5 y), 80 females (median age 9 y) and 5 quality assurance scheme specimens. Viruses were recovered from a number of specimen types including broncho-alveolar lavage, nasopharyngeal secretions, sputa, post-mortem lung tissue and combined throat and nasal swabs. Viral detection by IF was poor in sputa and respiratory swabs. A total of 99 viruses were detected in the study from 79 patients and 4 quality control specimens: 31 by immunofluorescence and 99 using the molecular strip. The strip consistently out-performed immunofluorescence with no loss of diagnostic specificity.

**Conclusions:**

The touchdown protocol with pre-dispensed primer master-mixes was suitable for replacing virus culture for the diagnosis of respiratory viruses which were negative by immunofluorescence. Results by immunofluorescence were available after an average of 4–12 hours while molecular strip results were available within 24 hours, considerably faster than viral culture. The combined strip and touchdown protocol proved to be a convenient and reliable method of testing for multiple viruses in a routine setting.

## Background

Acute respiratory tract infections are major causes of morbidity and mortality. In 2000, lower respiratory tract infections were globally the number one infectious cause of disability adjusted life-years [1]. The commonest respiratory viruses that cause acute upper and lower respiratory tract infections and which are routinely tested for in most virus diagnostic laboratories are: influenza A virus (FLA); influenza B virus (FLB); respiratory syncytial virus (RSV); parainfluenza virus type 1 (PF1); parainfluenza virus type 2 (PF2); parainfluenza virus type 3 (PF3) and adenovirus (ADV). Additionally, human rhinoviruses (HRV) and coronavirus 229E (CoV-229E) are also linked to acute respiratory infection but less commonly included in laboratory reports; human metapneumovirus (hMPV) is not yet part of most United Kingdom virus laboratory test repertoires (personal feed-back from the United Kingdom Clinical Virology Network).

As part of service development it was necessary to provide an alternative to virus culture for testing immunofluorescence negative respiratory specimens. Historically and indeed currently immunofluorescence [2] and virus culture [3,4] are the main methods used to diagnose acute respiratory virus infections. Culture is accepted as more sensitive than immunofluorescence but slower and therefore less useful for direct patient management decisions. Using a standard culture technique [4] for the culture of respiratory viruses our median reporting times for culture positive and culture negative specimens were 6 days (based on 407 specimens) and 7 days (based on 2159 specimens) respectively; virus identification by this technique required the use of monoclonal antibody staining of the cell monolayer in addition to observation for viral cytopathic effect. We therefore wished to develop a test capable of reporting on immunofluorescence negative specimens within a 24 hour period.

Increasingly however, the sensitivity of nucleic acid amplification techniques for diagnosis has become recognised [5-10]. However widespread concerns about contamination issues [11,12] and perceived cost [13] have slowed their widespread adoption. An added problem for acute respiratory tract infections is the relatively large number of viruses that need to be accounted for, a problem which presents specific technical challenges.

One such challenge is the different optimal annealing temperatures of the primer sets for each prospective virus target. The ABI PRISM 7000 real-time facility from Applied Biosystems addresses this by using bundled software to design primer/probe combinations that use a common amplification protocol. However this approach is compromised by the inability of software to allow for target heterogeneity. In addition it does not allow users to adopt clinically validated primer sets from the literature.

To address these problems we adopted an alternative approach through the development of a generic touchdown amplification protocol. Touchdown protocols involve a pre-designed stepped reduction in the annealing temperature used for primer-to-template binding, which introduces a competitive advantage for specific base-pair priming over non-specific priming [14]. A detailed knowledge of the optimum annealing temperature is therefore not required. The study protocol was empirically constructed and proved robust when applied to a large range of respiratory viral and bacterial targets, without compromising individual test sensitivity. It was designed for use with in-house primer master-mixes that recognise 12 common respiratory viruses.

Before deciding on the layout of the molecular strip, as described in the methods, we undertook a wide range of preliminary validation steps for each primer set. The complexity of the strip makes it impossible to fully evaluate using the classical approach of applying an individual gold standard to each virus type. Classically this approach works well where a single target is under investigation [15]. However although the strip is putatively designed to identify 12 viruses, the actual number of individual types targeted is over one hundred and sixty because of the inclusion of generic primer sets for HRV [16] and ADV [17] respectively. The classical approach is further compounded for viruses (a) that cannot be grown or grown easily; (b) for which commercial IF sera are not available; (c) for which specimen panels are not available. We therefore adopted a phased validation, culminating in the present study. Sensitivity was ascribed by undertaking copy number determination on cloned targets and these ranged form 6 × 10^3 ^copies per ml for human rhinovirus type 1b to 4.2 × 10^3 ^copies/ml for RSV-A. Specificity was ascribed through reproducibility, i.e. specimens which were repeatedly positive, following our standard clinical reporting algorithm [6], were regarded as true positives; a similar approach was recently described for hMPV [18]. In addition amplicon sequencing was used as an initial specificity check. The primers sets were tested on clinical respiratory specimens arising from a number of ethically approved studies. These included respiratory specimens from patients: (a) with chronic obstructive pulmonary disease; (b) with acute asthma; (c) on assisted ventilation in intensive care. They were also tested on respiratory specimens collected as part of an influenza spotter program as well as on laboratory specimens of known virus reactivity.

To test the feasibility of its routine use we needed to clinically validate its performance in a routine setting on specimens tested in parallel with our standard immunofluorescence protocol for the diagnosis of acute virus respiratory infections. Although the routine immunofluoresence panel lacked capacity for the detection of rhinoviruses, human metapneumovirus and CoV-229E, these were included on the strip for clinical reasons during the period of the study. These findings and their implications are reported.

## Results

### Patients and specimens

A total of 99 viruses were detected in 84/222 specimens from a total of 79/183 patients and 4/5 National External Quality Assurance Scheme (NEQAS) controls; immunofluorescence did not detect the parainfluenza virus type 2 virus in one of the NEQAS specimens. Viruses were detected in all of the specimen types processed. The molecular strip detected virus in: 16/36 (44.4%) broncho-alveolar lavages, 62/120 (51.6%) nasopharyngeal secretions, 11/35 (31.4%) sputa and 10/31 (32.2%) combined throat and nasal swabs. Immunofluorescence detected virus in: 6/36 (16.6%) broncho-alveolar lavages, 23/120 (19.1%) nasopharyngeal secretions, 1/35 (2.8%) sputa and 1/31 (3.2%) combined throat and nasal swabs.

The median age of male and female patients where virus was detected was 3 y (range 2 weeks – 79 years) and 4 y (5 weeks – 81 years) respectively. Sixteen viruses were detected in 14/27 (51.8%) specimens, confirming a respiratory virus in 12 out of 24 (50%) patients investigated in general practice. Seventy-nine viruses were detected in 70/191 (36.6%) specimens, confirming a respiratory virus in 67 out of 159 (42.1%) patients investigated in hospital. Of the 16 viruses detected in specimens from the community, PCR detected all 16 in contrast to a single identification, influenza A (H3), by immunofluorescence.

### Nested PCR

PCR identified one or more viruses in specimens from 84 of the 183 patients and the 4 NEQAS positive specimens, detecting a total of 99 viruses as shown in Table 1. The viruses detected were: influenza A (H3) virus (17); influenza A (H1) virus (4); influenza B virus (2); human rhinovirus (39); adenovirus (22); parainfluenza virus type 2 (1); parainfluenza virus type 3 (10); respiratory syncytial virus type A (2); respiratory syncytial virus type B (2);. No parainfluenza virus type 1, coronavirus 229E or human metapneumovirus were detected.

Dual infections were detected in 11/79 (13.9%) patients. The dual infections were: influenza A (H3) and adenovirus (4); influenza A (H3) virus and rhinovirus (2); influenza A (H1) and adenovirus (1); adenovirus and rhinovirus (3); respiratory syncytial virus type B and rhinovirus (1).

Nine patients had more than one specimen taken on the same day in which a virus was detected in at least one specimen by PCR. For 5 of the patients the same virus was detected in each of the 2 specimens. The viruses identified were rhinovirus (3), adenovirus (1) and parainfluenza type 3 (1); the latter was also immunofluorescence positive. In 2 cases a rhinovirus was detected in only one of the specimens. As part of a separate rhinovirus validation protocol one of these specimens was subjected to retesting coupled with limited sequencing of the 5' non-coding region amplicon which confirmed the presence of a rhinovirus sequence. Additionally, premature twin boys admitted to intensive care on the same day with severe bronchiolitis, both had evidence of acute rhinovirus infection by PCR. Limited sequencing of the 5' non-coding region of these viruses as part of the rhinovirus validation protocol confirmed the presence of an identical sequence of rhinovirus in both specimens.

### Immunofluorescence

Immunofluorescence identified a virus in specimens from 28 of the 183 patients and 3/4 NEQAS positive specimens (16.4%), detecting a total of 31 viruses as shown in Table 1. The viruses detected were: influenza A virus (15); influenza B virus (1); parainfluenza virus type 3 (8); respiratory syncytial virus (4); adenovirus (3). No parainfluenza virus types 1 or 2 were detected including a NEQAS mock parainfluenza virus type 2 infection which was recorded as negative. No dual infections were detected. One patient had 2 specimens taken on the same day in which the same virus, parainfluenza type 3, was detected.

## Discussion

Although touchdown PCR has been used successfully to help overcome some of the uncertainties associated with the thermal amplification of microbial nucleic acid targets [19-22], its use in this study has extended its role further and in so doing brought closer the goal of undertaking molecular diagnostics in a routine setting. Previously its main impact has been seen where multiplexing [23,24] or degenerate primers have been needed [25-27] and where the problems of choosing correct annealing temperatures are at their most difficult.

In this study the large number of targets is the main problem encountered. Using an empirical approach a series of amplification steps linked to a stepped reduction in annealing temperature from 55°C to 46°C was constructed. This proved extremely resilient when used with a wide range of primer sets and included the apparent anomaly of putting adenovirus through an initial reverse transcription step to stream line all of the targets on to a single strip; we have previously reported this approach for testing group F adenovirus alongside norovirus, astrovirus and rotavirus [28]. The touchdown surprisingly out-performed individual amplification protocols optimised for annealing temperature and thus proved suitable for use on the diverse range of respiratory viruses addressed in the study.

Where multiple viral targets are sought in clinical practice, we believe that it is only feasible to correlate the performance of the new assay in a head-to-head comparison with that already in routine use. Unfortunately for many clinical laboratories there is an elusion of testing for a wider range of viruses than is the case, by the inoculation of cell lines with a theoretical ability to grow the respective viruses. The annual reports of most clinical laboratories of one of the commonest human respiratory viruses, human rhinovirus, is an example of this; using the touchdown protocol we now report approximately 450 HRV infections per annum. The under reporting of adenovirus by standard methods [17] and the paucity of hMPV reporting, further underlines this assertion.

The ability to simultaneously validate the performance of multiple molecular primer sets in a routine clinical setting is a major accomplishment of the current methodological approach. The results demonstrated that a range of primers from both the medical literature and from in-house development could be amplified with a single generic touchdown protocol. It therefore confirmed the feasibility of directly incorporating primer sets into a standard operating procedure without the necessity for the individual optimisation of cycling parameters. As such the study results should facilitate primer selection and formal critical evaluation as here described. As an example of this enhanced flexibility we have recently replaced the primer sets for influenza A H1 and H3 (with respective copy number sensitivities of 8 × 10^3 ^and 2 × 10^3 ^copies per ml) with a generic matrix set (copy number sensitivity of 1 × 10^3 ^copies per ml).

The use of strips containing pre-dispensed mastermixes facilitates their use in a routine setting where laboratory personnel have only to thaw the strip and add the specimen extract. We make and aliquot for routine use a large range of multi-reaction mastermixes which are repeatedly subjected to freeze-thaw cycles as required on a daily basis. Provided the mixes are handled on ice, they remain extremely stable, over many months if so required. However the strip is designed for a single use only and thus only goes through a single freeze-thaw cycle. Mix stability is not a problem and the single positive control is used only to confirm that the touchdown amplification cycle has run successfully.

Because the technique of using nested amplification followed by running agarose gel electrophoresis is relatively cumbersome, it was important to evaluate how the complete protocol, inclusive of report generation, would perform when introduced into a routine line-managed diagnostic setting. Over the 18 months of the study the technique fitted in well to the demands of routine service. Central to this was the use of pre-dispensed and quality checked primer master-mixes which allowed the molecular strip to be adapted for use in a routine laboratory. The study confirmed that a broad based molecular approach was feasible as an alternative to virus culture to support immunofluorescence in the diagnosis of respiratory viruses. The overall superior performance of the strip and the missed NEQAS specimen by immunofluorescence underlines the need for a more sensitive back-up for negative specimens.

While nested protocols must be regarded as a pragmatic, interim solution until perfected single round systems are available, the format of the strip reduces the concern most attached to nested formats, i.e. false positive results. In our experience there is little evidence to support contamination arising from environmental sources and that the two major points of contamination in a nested system are (a) cross-contamination during manual extraction and (b) contamination of second round adjacent wells with product from a first round positive amplification. The use of the QIAGEN BioRobot for the extraction of all specimens reduced the former while the nature of the strip prevents the latter, since all the wells have separate mixes (Table 2). With both nested and non-nested assays the most critical requirements for reliable results are the use of well trained, appropriately skilled and knowledgeable staff, operating in a managed environment. As with any service, test performance must stand up to both external and internal quality assurance and in this regard we welcome the new respiratory quality control panel soon to be made available from Quality Control for Molecular Diagnostics (QCMD), Glasgow.

The results obtained were very encouraging. Although the strip was constructed to detect a wider range of viruses than immunofluorescence, over the period of validation it almost doubled (59 versus 31) the number of viruses that could have been detected by immunofluorescence, including a positive NEQAS specimen which was missed by immunofluorescence. Of this group of viruses the detection of adenovirus showed the most dramatic increase, an observation we have also previously made in a separate study [17] and which we continue to see both in routine respiratory specimens and in a number of respiratory studies. Similar to HRV viruses we believe these common infections are underdiagnosed by the standard techniques of immunofluorescence and culture. They are the second commonest virus, after HRV, that we observe in mixed infections and it is self-evident that these additional infections are at a level below the detection thresholds of standard methods. Their clinical significance when detected at these lower copy numbers remains to be determined.

As mentioned in the introduction a factor which often impacts negatively on a laboratory's decision to use molecular diagnostics is one of cost. It is worth considering that no matter which assay is chosen for use, it will attract the same overheads needed to provide the infrastructure of a laboratory set-up i.e. building, utilities, staff and equipment. In this regard there are no cheap tests and to use reagent costs as the sole factor in determining which assay to use is somewhat perverse. While the reagent costs of the strip are higher that commercial immunofluorescence reagents by a factor of 3, including extraction, this would undoubtedly narrow if immunofluorescence were capable of closing the pathogen gaps that currently exist e.g. HRV, hMPV. Currently using this approach, we have been able to replace both immunofluorescence and viral culture and this ability makes molecular diagnostics a more cost effective method for diagnosing viral infections. Taking into account the superior range, sensitivity, ability to quantify and speed of molecular techniques it is incredible how little they are used in routine laboratories. With the advent of SARS and the threat of avian influenza, this deficit is now beginning to disturb health care planners at the highest level.

Because specimen sampling was not contiguous seasonal peaks were not detected, accounting for the small numbers of respiratory syncytial virus detected and the lack of detection of human metapneumoviruses, parainfluenza virus type 1 and coronavirus 229E; subsequent (unpublished) data from the routine use of the molecular strip support an important role for human metapneumovirus in acute respiratory infections and the sporadic nature of infections caused by parainfluenza type 1 and coronavirus 229E.

Several interesting observations need highlighting. First, for immunofluorescence to perform reliably it was essential that a good nasopharyngeal specimen was available. The use of throat and/or nasal swabs with immunofluorescence alone is inappropriate. Second, immunofluorescence was very poor at detecting viruses from patients in the community, again almost certainly because of the universal use of swabs in that setting. Third, the rapid results of immunofluorescence were complemented by the touchdown protocol which can report definitive results within 24 hours, considerably faster than culture. Fourth, the molecular strip was better at detecting multiple infections. Even allowing for the inability of immunofluorescence to detect rhinoviruses, it should have detected the mixed adenovirus and influenza virus infections. Although immunofluorescence is capable of diagnosing dual infections, its routine use along with culture probably grossly underestimates their prevalence. The most plausible explanation is that the molecular technique detects infections where one of the viruses is below the detection threshold of immunofluorescence. These low level viruses are either just starting or more likely reaching the end of an infectious episode (latency is less likely) and this raises the previously unaddressed question of their role in viral respiratory pathogenesis. Fifth, the extent of rhinovirus infections was very significant. Their clinical significance ranged from acting as a definitive respiratory pathogen to a less certain role when acting as the most frequently detected co-pathogen in mixed infections.

## Conclusions

In conclusion the use of the touchdown protocol with pre-dispensed and quality checked primer master-mixes was suitable for replacing virus culture for the diagnosis of respiratory viruses for immunofluorescence negative specimens. Immunofluorescence results were available after an average of 4–12 hours while molecular strip results were available within 24 hours, considerably faster than viral culture. The combined strip and touchdown protocol is a convenient and reliable method of testing for multiple viruses in a routine setting. Its generic nature makes it especially useful for introducing test repertoire modifications e.g. incorporating primers for the newly identified coronaviruses SARS-CoV and HCoV-NL63.

## Methods

### Patients and specimens

A total of 222 specimens were included in the validation between January 2002 and June 2003, including 14 from an influenza surveillance scheme. The specimens were collected from 183 patients including: 103 male, median age 3.5 y (7 m – 84 y); 80 female patients, median age 9 y (7 m – 84 y); both male and female ages were skewed towards the lower age ranges, and 5 national external quality assurance scheme (NEQAS) specimens (4 positive, 1 negative). One hundred and fifty-nine patients were in hospital and 24 were in the community at the time of sampling. Specimens tested consisted of a wide range of specimens including: broncho-alveolar lavage (36), nasopharyngeal secretions (120), sputum (35) and combined throat and nasal swabs (31).

### Immunofluorescence

Nasopharyngeal secretions, broncho-alveolar lavage and sputum specimens were received in dry sterile containers at ambient temperature. Upon receipt they were re-suspended in 2 ml of virus transport medium (VTM) consisting of phosphate buffered saline pH 7.1, bovine serum albumin 7.5 μg/ml, penicillin G sodium 1000 units/ml, streptomycin sulphate 1000 μg/ml and amphotericin B 2.5 μg/ml. Throat and nasal swabs were received in 2 ml of VTM and vortexed on arrival to release cells attached to the fibres of the swab. An aliquot of 410 μl was taken off for extraction after which the specimens were centrifuged at 2600 g for 5 min and the resulting cell deposits air-dried on glass multi-well slides and fixed in acetone prior to testing. Immunofluorescence was set up on the respiratory specimens using commercial reagents according to the manufacturer's instructions, and was able to detect: influenza A, influenza B, respiratory syncytial virus, adenovirus, parainfluenza type 1, parainfluenza type 2 and parainfluenza type 3 (Dako diagnostics, Ely, UK).

### Specimen extraction

A volume of 200 μl of the respiratory specimen suspension was extracted on a QIAGEN BioRobot 9604 using the Blood and Body Fluid Vacuum Protocol of the QIAamp DNA Blood Kit (Qiagen Ltd., Crawley, England, U.K). This protocol allows the co-extraction of both RNA and DNA simultaneously.

### Nested PCR

Simultaneous amplification of all targets was facilitated by using a standard 8 well multi-well PCR strip to which all mixes were pre-dispensed and stored frozen; this format is referred to in the paper as the "respiratory strip" because of the respiratory nature of the targets. The respiratory strip targeted the following 12 common respiratory viruses: influenza A (H3), influenza A (H1), influenza B, respiratory syncytial virus type A, respiratory syncytial virus type B, adenovirus, coronavirus 229E, parainfluenza virus type 1, parainfluenza virus type 2, parainfluenza virus type 3, human rhinovirus and human metapneumovirus. The final configuration of the single and multiplex primer mixes in the 8 well strip are shown in Table 2. The primer sets used were taken mainly from published studies [16,29-31] but also included primer sets validated in-house after modification or de-novo design, including those for influenza A (H1), influenza A (H3) and the generic adenovirus primers [17]. The primers, gene targets and expected product sizes following amplification are shown in Table 3.

Each primer master-mix was made-up and titrated against a known positive control before being aliquoted and dispensed into its respective well of the 8-well microtube strip. The strips were stored frozen at -20°C until used. A positive control was also aliquoted and stored separately at -20°C until used. For the duration of the study the positive control was the cloned target of parainfluenza virus type 1; a negative control was not deemed necessary.

First round volumes were made-up in Access RT-PCR buffer (Promega, Southampton, England, U.K) and in the final 10 μl volume contained the following reagent amounts: 1.5 mM MgSO_4_, 1 unit AMV reverse transcriptase, 1 unit Tfl DNA polymerase, 0.2 mM each deoxynucleoside triphosphate (dATP, dCTP, dGTP, dTTP) and 1 μM outer primers.

Second round volumes were made-up in Taq Buffer B (Promega) and in the final 10 μl volume contained the following final amounts: 10 mM Tris-HCl (pH 9.0), 3.5 mM MgCl_2_, 50 mM KCl, 0.1% Triton X-100, 0.2 mM of each deoxynucleoside triphosphate (dATP, dCTP, dGTP, dTTP), 0.25 units of Taq DNA polymerase (Promega) and 0.2 μM inner primers.

First round amplification was performed on 2 μl of extract added to 8 μl of first round primer master-mix per well. Second round amplification was performed on 0.2 μl of the first round reaction added to 9.8 μl of second round primer master-mix per well; a multi-channel pipette facilitated the transfer of the 8 volumes in one step. The positive control was run on the eighth well of each strip. The second round products were run on ethidium bromide stained 2% agarose gels and photographed. Specimens were reported positive when respectively the correct size bands and the positive control bands were present.

### Touchdown amplification protocol

Amplification was carried out on a range of thermal cyclers including the Applied Biosystems GeneAmp 2400 and 9700 series and a DNA Engine Tetrad PTC 225 (MJ Research, USA). The first and second round amplification protocols consisted of 36 identical cycles with the exception that (a) a reverse transcription step of 48°C-10 min preceded the first round and (b) a hot-start preceded the second round by transferring the strip directly from ice to a thermal cycler held at 94°C. The touchdown protocol consisted of 6 steps as follows: (a) initial denaturation (94°C-2 min); (b) 3 cycles of denaturation (94°C-30 s), annealing (55°C-30 s) and extension (72°C-30 s); (c) 3 cycles of denaturation (94°C-30 s), annealing (52°C-30 s) and extension (72°C-30 s); (d) 20 cycles of denaturation (94°C-30 s), annealing (49°C-30 s) and extension (72°C-30 s); (e) 10 cycles of denaturation (94°C-30 s), annealing (46°C-30 s) and extension (72°C-30 s); (f) 72°C for 5 mins.

## Authors' contributions

PVC: Touchdown and molecular strip design and manuscript preparation.

GMO: Early application of touchdown cycling to respiratory samples.

HJO'N: Study protocol and manuscript preparation.

CMcC: Study protocol and manuscript preparation.

DDEO: Routine application of touchdown protocol during study.

FM: Routine application of touchdown protocol during study.

SJM: Routine application of touchdown protocol during study.

SAF: Primer-mastermix manufacture and quality control.

DEW: Early application of touchdown cycling to respiratory samples and manuscript preparation.

MF: Routine application of immunofluorescence protocol during study.

JS: Design and validation of primers for human metapneumonavirus detection.

## Figures and Tables

**Table 1:** Viruses detected by the molecular strip and immunofluorescence.

Virus	Molecular Strip	Immunofluorescence
Influenza A Virus	21*	15
Influenza B Virus	2	1
Human Rhinovirus	39	Not Tested
Adenovirus	22	3
Parainfluenza Virus Type 2	1	0
Parainfluenza Virus Type 3	10	8
Respiratory Syncytial Virus	4^§^	4
Total Positive	99	31
Total Tested	222	222

* Included 17 influenza A H3 and 4 influenza A H1.

^§ ^Included 2 respectively of respiratory syncytial virus type A and type B.

**Table 2:** Template for position of primer master-mixes on the molecular strip.

Well Number	Virus
1	Parainfluenza Virus Types 1,2,3
2	Human metapneumovirus
3	Influenza A H1 & oronavirus 229E
4	Influenza A H3 & Human Rhinovirus
5	Influenza B Virus
6	Respiratory Syncytial Virus Types A & B
7	Adenovirus
8	Positive Control

**Table 3:** Primer sequences, gene targets and expected product sizes for viruses on the molecular strip.

Name	Sequence (5' to 3')	Gene Target	1^st^/2^nd^	Size (bp)
PF1 1A	CCT TAA ATT CAG ATA TGT AT	HN^1^	1	478
PF1 1B	GAT AAA TAA TTA TTG ATA CG			
PF1 1C	CCG GTA ATT TCT CAT ACC TAT G		2	317
PF1 1D	CCT TGG AGC CGA GTT GTT AAG			
PF2 1A	AAC AAT CTG CTG CAG CAT TT	HN^1^	1	508
PF2 1B	ATG TCA GAC AAT GGG CAA AT			
PF2 1C	CCA TTT ACC TAA GTG ATG GAA T		2	204
PF2 1D	GCC CTG TTG TAT TTG GAA GAG A			
PF3 2A	CTTG TAA ACT CAG ACT TGG TA	HN^1^	1	478
PF3 2B	TTT AAG CCC TTG TCA ACA AC			
PF3 2C	ACT CCC AAA GTT GAT GAA AGA T		2	103
PF3 2D	TAA ATC TTG TTG TTG AGA TTG			
hMPV 1A	GCG GCA ATT TTC AGA CAA CG	Fusion	1	696
hMPV 1B	ACA TGC TGT TCG CCT TCA AC			
hMPV 1C	CAG CAG CAG GAA TCA ATG TT		2	288
hMPV 1D	TCG CCT TCA ACT TTG CTT AG			
CoV229E 1A	GGT ACT CCT AAG CCT TCT C G	Nucleocapsid	1	450
CoV229E 1B	TGC ACT AGG GTT AAG AAG AGG			
CoV229E 1C	TTT GGA AGT GCA GGT GTT GTG G		2	100
CoV229E 1D	GAC TAT CAA ACA GCA TAG CAG C			
ENT 2A	TCC TCC GGC CCC TGA ATG	5' Non-coding	1	120
ENT 2B	GAA ACA CGG ACA CCC AAA GTA			
ENT 1C	GGC CCC TGA ATG CGG CTA AT		2	90
HRV 1B	GGT CCC RTC CCG CAA TT			
FLA 3A (H1)^2^	GAA ATT TGC TAT GGC TGA C	Haemagglutinin	1	482
FLA 3B (H1)^2^	ATA TTT TGG GCA CTC TCC TAT			
FLA 3C (H1)^2^	GTC TCT GTA GTG TCT TCA CAT TAT		2	197
FLA 3D (H1)^2^	CCG GAC CCA AAC CCT CTA CTC			
FLB 1A	GTG ACT GGT GTG ATA CCA CT	Haemagglutinin	1	900
FLB 1B	TGT TTT CAC CCA TAT TGG GC			
FLB 1C	CAT TTT GCA AAT CTC AAA GG		2	767
FLB 1D	TGG AGG CAA TCT GCT TCA CC			
FLA 2C (H3)^2^	AGC AAA GCT TTC AGC AAC TG	Haemagglutinin	1	591
FLA 2D (H3)^2^	GCT TCC ATT TGG AGT GAT GC-3			
FLA 2E (H3)^2^	AGT GCT GAA CGT GAC TAT GC		2	149
FLA 2F (H3)^2^	TTT GCT GGC TTC TCT TGG T			
RSV 2A	GTC TTA CAG CCG TGA TTA GG	Nucleoprotein	1	838
RSV 2B	GGG CTT TCT TTG GTT ACT TC			
RSVA 2C	GAT GTT ACG GTG GGG AGT CT		2	334
RSVA 2D	GTA CAC TGT AGT TAA TCA CA			
RSVB 2C	AAT GCT AAG ATG GGG AGTTC		2	183
RSVB 2D	GAA ATT GAG TTA ATG ACA GC			
ADV 2A^2^	GCCGCAGTGGTCTTACATGCACATC	Hexon	1	301
ADV 2B^2^	CAGCACGCCGCGGATGTCAAAGT			
ADV 2C3^2^	GACGCCTCGGAGTACCTSWSYCC		2	185
ADV 2DD^2^	TACGAGTACGTGGTGTCCTCKCGRTC			

^1 ^HN = Haemagglutinin-neuraminidase gene; ^2 ^Primer sets designed for this study. N.B. 1 = 1^st ^round primer set, 2 = 2^nd ^round primer set; ENT 2A 2B, 1C, HRV 1B primer set = Rhinovirus.
